# Pyrexia of Foreign Origin: Exploring the Impact of Travel History

**DOI:** 10.7759/cureus.74279

**Published:** 2024-11-22

**Authors:** Justin Kadamala Samuel, Sukhvinder S Digpal, Farzia Tanzum

**Affiliations:** 1 Internal Medicine, Portsmouth Hospitals University, Portsmouth, GBR; 2 Clinical Research, Amrita Institure of Medical Sciences, Kochi, IND; 3 Internal Medicine, Portsmouth Hospitals University NHS Trust, Portsmouth, GBR

**Keywords:** endemic infections, falciparum malaria, history-taking, infectious disease medicine, toxicology and tropical medicine

## Abstract

Malaria remains a significant global health challenge, particularly in endemic regions of Africa, with *Plasmodium falciparum* being the most virulent species. This case report details the presentation of a 24-year-old Caucasian woman who collapsed at a train station in the United Kingdom after experiencing a week of fever, malaise, abdominal pain, and gastrointestinal symptoms. At emergency care, she was initially resuscitated with intravenous fluids and antipyretics. Laboratory investigations indicated a normal hemoglobin level but a markedly low platelet count. Initial differentials included sepsis, viral infection, and idiopathic thrombocytopenic purpura. Following her transfer to the medical ward, her travel history elicited a month-long trip to Nigeria. A malaria screen subsequently tested positive for *P. falciparum*, leading to the initiation of intravenous artesunate. The diagnosis was confirmed through thick and thin blood films, and treatment was adjusted to oral artemether and lumefantrine. The patient showed significant clinical improvement, with normalization of platelet counts and a decrease in inflammatory markers. This case underscores the importance of thorough history-taking, particularly regarding travel to endemic areas, for the timely diagnosis of malaria. It highlights the necessity for healthcare professionals to maintain a high index of suspicion for malaria in returning travelers presenting with fever, regardless of any prophylactic measures taken. The report emphasizes the critical need for awareness and education surrounding malaria, especially in non-endemic regions, to prevent severe complications and improve patient outcomes.

## Introduction

Malaria is a significant parasitic infection that predominantly affects regions in Africa. With approximately 300 million cases reported annually, the disease claims the lives of one to two million individuals each year [1]. Among the four species of malaria parasites that infect humans, *Plasmodium falciparum* is notorious for causing severe morbidity and mortality, particularly in vulnerable populations like children and pregnant women [2]. Despite the implementation of various strategies, such as community health outreach and the distribution of insecticide-treated nets, the impact on malaria incidence has been limited.

## Case presentation

A 24-year-old Caucasian woman was brought to the emergency department (ED) at Portsmouth Hospitals University, United Kingdom, after collapsing at a train station. She reported a three-day history of fever, malaise, and myalgia. Additionally, she had abdominal pain, nausea, vomiting, night sweats, and dizziness for the past week. She worked as an interior designer. Her medical history was unremarkable. She was a non-smoker and non-alcoholic.

Initial examination and findings

Upon examination, the patient was febrile with a temperature of 38.5°C, blood pressure of 85/50 mmHg, heart rate of 100 bpm, respiratory rate of 15 breaths per minute, and oxygen saturation of 96% on room air. The general examination revealed no signs of meningism or neurological deficits, and chest and abdominal examinations were unremarkable. Initial resuscitation included intravenous fluids and antipyretics.

Laboratory Investigations were conducted, including a full blood count, biochemistry, and liver function tests. The initial full blood count indicated a normal hemoglobin level but a significantly reduced platelet count of 48 (Table 1).

**Table 1:** Hematology and biochemical values before and after treatment

Parameters with range	On admission	On discharge
Hemoglobin (120-150 g/L)	132	11
White cell count (4.0-11.0 × 10^9^/L)	4.1	6.1
Platelets (150-410 × 10^9^/L)	48	328
CRP (0-7 mg/L)	93	1
Total bilirubin (0-21 µmol/L)	15	11
ALP (30-130 U/L)	94	93
Hematocrit (0.360-0.460 L/L)	0.39	0.35
Creatinine (45-84 µmol/L)	65	64
Sodium (133-146 mmol/L)	130	141

Blood and urine cultures sent as part of a septic screen showed no growth (Table 2), while chest and abdominal X-rays were reported as normal.

**Table 2:** Urine and blood culture reports showing no growth

Specimen	Report
Mid-stream urine	White blood cells: 1-50, epithelial cells: NOT seen. Culture values do not meet the threshold of significance at which culture is indicated.
Peripheral blood culture	Aerobic and anaerobic culture sterile at day 5.

Differential diagnoses

At this point, differential diagnoses included viral illness, immune thrombocytopenic purpura (ITP), and collapse secondary to dehydration. Antibiotics were initiated to cover potential bacterial infections.

The patient was transferred under medics, and a thorough history and examination were conducted by the medical team in the acute medical ward. The patient revealed that she had recently traveled to Nigeria for one month, after which she developed abdominal pain, nausea, intermittent vomiting, and dizziness upon returning to the United Kingdom a week prior. She also did not take malaria prophylaxis before travel. This travel history was crucial in guiding the diagnosis. The patient's symptoms of cyclical fevers, night sweats, and malaise combined with her travel history raised suspicion for malaria.

Diagnosis and treatment

**Figure 1:**
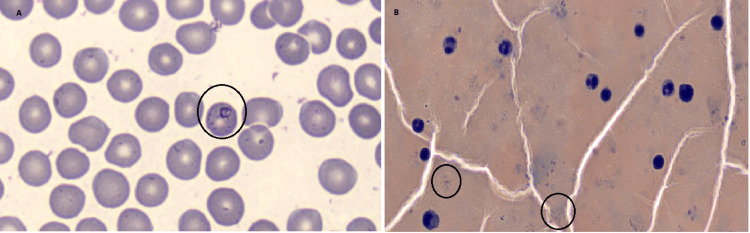
(A) Thin slide of Plasmodium falciparum. (B) Thick slide of Plasmodium falciparum.

A malaria antigen test was positive for *P. falciparum*/mixed. The involvement of infectious disease specialists and microbiologists led to the initiation of intravenous artesunate while awaiting further confirmatory tests. The subsequent malarial thick and thin film confirmed the presence of *P. falciparum *with a parasite count of 0.5%-0.8% (Table 3 and Figure 1, Panels A and B), prompting a switch to oral artemether and lumefantrine. Blood and urine cultures were negative, leading to the cessation of antibiotics. The patient’s condition improved, evidenced by a downtrend in CRP and normalization of platelet counts (Table 1).

**Table 3:** Diagnostic tests for malaria RDT: Rapid diagnostic test.

Malarial tests	Results
Malaria antigen test	RDT positive for *P. Falciparum* or mixed
Thick film	Malarial parasites are present
Thin film	Ring form malarial parasites seen, suspicious for *Falciparum *malaria - parasitemia = 0.5%-0.8%

## Discussion

Malaria is the most frequently imported tropical infection in the United Kingdom [3]. In 2023, 2,004 malaria cases were reported, an increase compared to the previous year, of which 1,369 were imported [4]. This highlights the importance of vigilance in recognizing malaria in patients with a history of travel to endemic regions, further emphasizing the significance of a good travel history.

Tests using blood films for malaria in all travelers returning from the tropics with a fever and thick and thin blood films are the gold standard investigation [5]. To exclude malaria, three negative diagnostic samples, each taken 12-24 hours apart, are necessary. The Hematology laboratory will routinely process an antigen dipstick test when a malaria blood film is requested [4]. Any uncertainty over infecting species should be treated as *Falciparum *malaria.

All patients diagnosed with *Falciparum *malaria require admission for a minimum of 24 hours for observation. The severity of the infection is based upon a parasitemia of >2% and/or complicating clinical features: conscious level, acidosis, pulmonary edema, renal failure, anemia, and hypoglycemia [6].

In severe malaria or emergency cases, intravenous artesunate is the treatment of choice. Intravenous quinine can be commenced as an alternative until artesunate is available. Patients with severe *Falciparum *malaria can deteriorate rapidly, so early intensive care input would be prudent [6].

Patients diagnosed with *Falciparum *malaria usually present within one month of returning from the tropics, although 10% of cases can present up to three months after travel. Uncomplicated *Falciparum* malaria and non-*Falciparum *malaria should be treated with artemether and lumefantrine, and all patients should be referred to the local infectious disease team for monitoring and advice.

Malaria can rapidly progress to a severe and life-threatening illness, if not treated promptly. Assessing patient severity based on parasitemia will guide improved outcomes and will change treatment management.

It is essential to suspect malaria in any patient presenting with fever or a history of fever who has traveled to a malaria-endemic region, regardless of antimalarial prophylaxis [6]. A combination of artemether with lumefantrine is the recommended first-line treatment for uncomplicated *P. falciparum *malaria [6].

## Conclusions

In conclusion, the case of this 24-year-old woman emphasizes the critical role of thorough history-taking at presentation. By eliciting travel history and recognizing the symptoms of malaria, healthcare professionals can initiate timely diagnosis and treatment, ultimately preventing severe complications associated with this potentially life-threatening disease. Emphasizing the importance of malarial prophylaxis before traveling to endemic countries is essential. Although malaria is uncommon in the United Kingdom now, cases continue to rise. Awareness and education about the disease remain paramount in safeguarding public health.
